# Differential Response Following Infection of Mouse CNS with Virulent and Attenuated Vaccinia Virus Strains

**DOI:** 10.3390/vaccines7010019

**Published:** 2019-02-12

**Authors:** Tomer Israely, Nir Paran, Noam Erez, Lilach Cherry, Hadas Tamir, Hagit Achdout, Boaz Politi, Ofir Israeli, Galia Zaide, Inbar Cohen-Gihon, Einat B. Vitner, Shlomo Lustig, Sharon Melamed

**Affiliations:** 1 Department of Infectious Diseases, Israel Institute of Biological Research (IIBR), Ness-Ziona, Israel; tomeri@iibr.gov.il (T.I.); nirp@iibr.gov.il (N.P.); noame@iibr.gov.il (N.E.); Lilachc@iibr.gov.il (L.C.); hadast@iibr.gov.il (H.T.); hagita@iibr.gov.il (H.A.); boazp@iibr.gov.il (B.P.); einatv@iibr.gov.il (E.B.V.); shlomolustig@gmail.com (S.L.); 2 Department of Biochemistry and Molecular Genetics, Israel Institute of Biological Research (IIBR), Ness-Ziona, Israel; ofiri@iibr.gov.il (O.I.); galiaz@iibr.gov.il (G.Z.); inbarg@iibr.gov.il (I.C.-G.)

**Keywords:** vaccinia, brain, meningoencephalitis, smallpox, neurovirulence, RNA-seq

## Abstract

Viral infections of the central nervous system (CNS) lead to a broad range of pathologies. CNS infections with Orthopox viruses have been mainly documented as an adverse reaction to smallpox vaccination with vaccinia virus. To date, there is insufficient data regarding the mechanisms underlying pathological viral replication or viral clearance. Therefore, informed risk assessment of vaccine adverse reactions or outcome prediction is limited. This work applied a model of viral infection of the CNS, comparing neurovirulent with attenuated strains. We followed various parameters along the disease and correlated viral load, morbidity, and mortality with tissue integrity, innate and adaptive immune response and functionality of the blood–brain barrier. Combining these data with whole brain RNA-seq analysis performed at different time points indicated that neurovirulence is associated with host immune silencing followed by induction of tissue damage-specific pathways. In contrast, brain infection with attenuated strains resulted in rapid and robust induction of innate and adaptive protective immunity, followed by viral clearance and recovery. This study significantly improves our understanding of the mechanisms and processes determining the consequence of viral CNS infection and highlights potential biomarkers associated with such outcomes.

## 1. Introduction

Viral infection of the central nervous system (CNS) is associated with a broad spectrum of clinical manifestations ranging from asymptomatic to lethal disease. The blood–brain barrier (BBB) protects the CNS from invading pathogens by controlling exchange between the blood, the CNS, and the immune system. Components of the immune system that have a role in controlling viral infections of the CNS include the innate and adaptive immune systems and local, brain-resident immune components. Viruses and bacteria have developed different strategies to evade these obstacles and penetrate the brain [1]. Viruses entering the CNS belong to different virus families including Rhabdoviridae (e.g., Rabies virus), Flaviviridae (e.g., West-Nile virus, Zika virus, and Tick-borne encephalitis virus), Alphaviruses (e.g., Semliky forest virus, Sindbis virus, and Venezuelan equine encephalitis virus), Alpha herpesviruses, and others. Vaccinia Virus (VACV) belong to the Orthopoxviridae family of large DNA enveloped viruses. Variola virus (VARV) and Monkeypox virus (MPXV) are two examples of Orthopoxviruses causing smallpox and monkeypox, respectively, two devastating human diseases. Although poxviruses are not considered typical encephalitic or meningitic viruses, in certain circumstances, they can penetrate and infect the CNS. Historical anecdotal reports indicated that in addition to the known manifestations of smallpox, there are indications of severe encephalomyelitis following VARV infection of the CNS [2,3,4]. Previous reports indicate that in certain rare circumstances encephalitis developed following smallpox vaccination, considered as postvaccinal encephalitis [5,6,7,8]. Although smallpox has been eradicated from nature, the potential risk of its reemergence through accidental or intentional release calls for an increased interest in the disease and its countermeasures (vaccines and antivirals).

Smallpox eradication was achieved by immunization with different VACV strains including VACV-Lister and the New York City Board of Health (NYCBH) [3,9]. The vaccine based on the NYCBH strain of VACV was manufactured by Wyeth Laboratories, Inc. and designated Dryvax^®^ or VACV-Wyeth. These heterogeneous vaccinia virus strains were used, among others, during the worldwide eradication campaign. Following 18 intracerebral passages of the VACV-Wyeth strain in mice, VACV-Western Reserve (VACV-WR) was isolated [10] and since then has served as a common laboratory strain. In subsequent studies, VACV-WR was found to be virulent to immunocompetent mice following intracranial (i.c.) and intranasal (i.n.) administration in various mouse strains [11,12,13,14,15]. Comparison between VACV-WR and VACV-Wyeth following an intranasal challenge in C57BL/6 mice revealed local higher viral replication and immune suppression by VACV-WR compared to VACV-Wyeth [16]. However, a comparison between these viruses in the context of neurovirulence (by direct inoculation) is missing.

To elaborate on the differences between the vaccine attenuated strains VACV-Lister and VACV-Wyeth and the virulent VACV-WR strain following brain infection, mice were infected i.c. and followed by kinetic multi parametric analysis of both viral and host response to the infection. Intracranial infection can be used to address questions like virulence/attenuation and neuroadaptation and has also been used to demonstrate the attenuation of the vaccine strains [16,17,18,19,20,21]. Direct i.c. infection allowed us to focus on the events following infection and to exclude possible differences between strain replication in peripheral tissues. This may include migration to and penetration through the BBB and differences in the host immune response before entering the brain. There is a need to understand the host response to viral replication in the CNS at both asymptomatic and symptomatic stages, mainly, the differential response following pathological replication versus acute protective response. Such data would help in identification of clinical prognostic markers, to search for new therapeutic targets and as an established model for the evaluation of vaccine safety and potency, drug′s efficacy and to gain further knowledge on the viral mechanisms of pathogenicity and immune evasion. 

In this study, we show that at early post-infection (p.i.) stages, during the asymptomatic incubation period, VACV-WR exerted robust immune silencing within the CNS that coincided with robust virus replication. At advanced stages of the disease, significant morbidity was accompanied by robust viral replication, increased BBB permeability and spreading to other tissues. At this point, antiviral immunity was evident, yet mice succumbed to the infection. In sharp contrast, brain infection with an attenuated vaccine strain was followed by early and robust induction of robust antiviral immunity in the brain, viral clearance and recovery. In this work, we followed these processes at multiple levels including host gene expression, viral replication, immune response and tissue pathological analyses.

## 2. Materials and Methods

### 2.1. Cells and Viruses

Vero (ATCC CCL-81) and BS-C-1 (ATCC CCL-26) cells were used and maintained by routine methods as recommended by the ATCC. VACV-Lister (Elstree; provided by the Israeli Ministry of Health) was tittered on Vero cells as described previously [22]. VACV-WR (ATCC VR-119), recombinant VACV-WR (VACV-WRvFire), and Wyeth New York City Board of Health (VACV-WyethvFire) expressing firefly luciferase via synthetic early-late promoter (kindly provided by Prof. B. Moss (Bernard Moss, Laboratory of Viral Diseases, National Institute of Allergy and Infectious Diseases, National Institutes of Health, Bethesda, MD, USA); [23,24]) were tittered on BS-C-1 cells.

### 2.2. Animal Experiments

Female BALB/c mice (5–8 weeks old) were purchased from Charles River Laboratories, UK. For i.c. viral administration, mice were anesthetized (Ketamine 75 mg/kg, Xylazine 7.5 mg/kg in phosphate buffered saline (PBS)) and injected with 30 μL of the examined virus diluted in PBS + 2% heat-inactivated fetal calf serum (PBF). Diluted virus or the carrier buffer alone were injected using 27 G needle into the right frontal cerebral hemisphere. Penetration of the needle was restricted to a depth of 2 to 3 mm. General procedures for animal care and housing were followed in compliance with the regulations for animal experiments at the Israel Institute for Biological Research (IIBR). All experiment protocols were approved by the IACUC (Institutional Animal and Care Committee), of IIBR before the commencement of the studies (IACUC-IIBR M45-07 (approved on 21/10/2007), IACUC-IIBR M33-08 (approved on 07/10/2008), IACUC-IIBR M17-10 (approved on 12/04/2010), and IACUC-IIBR M71-16 (approved on 09/10/2016)). 

### 2.3. Determination of Viral Load in Mouse Organs

Viral loads were determined at the indicated days post-infection (d.p.i.). Mice were bled from the tail vein, anesthetized, and perfused with PBS via the heart before organ removal to liquid nitrogen and storage in −80 °C until further processing. Brain and spleen were processed for titration in 1.5 mL of ice-cold PBS as described previously [25]. Part of the processed tissue samples was used immediately for RNA extraction while the other part was kept in −80 °C until further processing for viral titration. Samples of VACV-Lister infected mice were tittered on Vero cells (ATCC CCL-81) while VACV-WR, VACV-WRvFire, and VACV-Wyeth were tittered on BS-C-1 cells (ATCC CCL-26).

### 2.4. Bioluminescence Imaging

Live imaging was performed with an IVIS Lumina II system (PerkinElmer, Waltham, MA, USA). D-Luciferin substrate (PerkinElmer, Waltham, MA, USA) was injected intraperitoneally (i.p.) (150 mg/kg body weight) 7 min before imaging. Mice were imaged once at two, four or five d.p.i or twice at days 7 and 11 or 9 and 13 under anesthesia (Ketamine 75 mg/kg, Xylazine 7.5 mg/kg in PBS; five mice per group). Images were collected along 1 s or 40 s with a binning factor of 4. The same region of interest (ROI) was used in all examined mice for the calculation of signal intensity from the head area. Light emission was measured in photons/s/cm^2^/sr (photon flux). Acquisition and analysis were performed with Living Image Software, Version 4.2 (PerkinElmer, Waltham, MA, USA). The signal to noise ratio of signal intensity from the head of each mouse over the background was calculated by division of the head signal intensity with the background signal intensity.

### 2.5. Histology and Immunohistochemistry

Mice at 5 d.p.i. were anesthetized and perfused with 4% paraformaldehyde (PFA) via the heart. Brains were retrieved for further fixation in 4% PFA for 7 to 14 days and transferred to 70% ethanol until further processing. Brains were sectioned to investigate the cerebrum/cortex, midbrain, hippocampus, brain stem and cerebellum. Paraffin-embedded tissue sections (5 μm) were stained with Hematoxylin and Eosin (H&E). For immunohistochemistry (IHC), slides were treated with One-Step HRP Polymer IHC kit anti-Rabbit or anti-Mouse and Rat IgG (H+L) (biotin free) (Immuno Bio Science Corp. Mukilteo, WA, USA). Antigen retrieval was done with 10 mM citrate buffer, 0.05% Tween-20, pH 6.0 (DB Biotech, Inc., Kosice, Slovak Republic) for 15 min in TintoRetriever Pressure Cooker, Heat Retrieval System (Bio SB, Santa Barbara, CA, USA). The virus was stained by in-house rabbit anti-VACV or mouse anti-VACV polyclonal antibody. Macrophages, and microglial cells were detected with monoclonal rabbit anti-iba-1 monoclonal antibody (Abcam). IHC detection was performed with 3,3′-Diaminobenzidine (DAB) chromogen substrate (Vector Laboratories Inc., Burlingame, CA, USA). 

Histopathological evaluation: To ensure equivocal and unbiased evaluation all H&E and IHC slide examination and scoring were evaluated by the same pathologist who was aware of the general experimental setup. Three brains infected with VACV-WR were compared to three brains infected with VACV-Wyeth, and one brain of PBF injected mouse 5 d.p.i. An overall brain inflammation score and the percentage of various cells were evaluated from the H&E stains. Scoring of the inflammatory grade was: no inflammation at all (score = 0); very mild inflammation (<10 cells per ×20; score = 1); mild inflammation (>10 to <20 cells per ×20; score = 2); moderate inflammation (>20 to <50 cells per ×20; score = 3); severe inflammation (>50 cells per ×20; score = 4). For the semi-quantitative evaluations of the IHC, 10 fields of ×20 objective magnification were evaluated per region in the dorsal hippocampus cross sections (cortex + meninges, corpus callosum, hippocampus, lateral ventricles, thalamus, hypothalamus). Scoring of the IHC grade for VACV positive cells was: no positive cells at all (score = 0); very few positive cells (<10 cells per ×20; score = 1); few positive cells (>10 to <20 cells per ×20; score = 2); moderate number of positive cells (>20 to <50 cells per ×20; score = 3); high number of positive cells (>50 cells per ×20; score = 4). 

### 2.6. Gene Expression

Total RNA samples (2, 4, and 5 d.p.i) from the homogenized tissues used for determination of viral load in mouse organs procedure described above, were extracted using RNeasy Mini Kit (Qiagen, Hilden, Germany) according to the manufacturer’s instructions. The extracted total RNA was quantified on a NanoDrop 100 spectrophotometer (Thermo Fisher Scientific, Waltham, MA, USA). The RNA quality was assessed using Bioanlyzer with the RNA high sensitivity kit (Aligent, USA). For all the samples, RNA integrity number (RIN) was calculated and three samples/group/time point with RIN value of >7.0 were sent to Columbia Genome Center (New York, NY, USA). Libraries were constructed using the TruSeq RNA library preparation kit (Illumina), and a whole transcriptome sequencing (total RNA-seq) using an Illumina HiSeq was performed. For each sample over 30 million single 100 nt reads were generated. DEseq files were constructed by a comparison of the data of the virally infected mice with the corresponding carrier buffer control of the same time point for log2 fold change and *p*-values. The log2 fold change was converted to fold change values in some of the cases for simplicity.

Functional analysis: Ingenuity Pathway Analysis (IPA^®^; Qiagen Redwood City, CA, USA) was used to evaluate the influence of the viral infection on biological canonical pathways and networks. IPA cutoff criteria for input list of differentially expressed genes was set to log2 fold-change of > ±1.3 and adjusted *p*-value of <0.05. Graphical representation of networks with their scores was computed by IPA. The nodes within the networks represent genes and lines indicate biological relationships (direct or indirect) with other genes based on published literature within the IPA software. The network score is based on hypergeometric distribution and is calculated with the right-tailed Fisher’s exact test.

### 2.7. Quantitative Real-Time RT-PCR

Total RNA extracted from brains infected with VACV-WR or VACV-Wyeth at 2, 4, and 5 d.p.i was used to measure changes in the transcripts of selected differentiating genes by qRT-PCR using the corresponding specific primers printed on 96 well plates (Custom TaqMan Array Plates, Applied Biosystems^TM^). The genes were divided to early (2 d.p.i.) and late (4–5 d.p.i.) differentiating genes. Information regarding the selected genes is presented in Supplementary Table S1. Briefly, 1 microgram cDNA was synthesized out of the RNA using Verso cDNA Synthesis Kit (Thermo Fisher Scientific, Waltham, MA, USA) according to the manufacturer’s instructions. Samples were subjected to qPCR with TaqMan^®^ Fast Advanced Master Mix (7500 Real Time PCR System, Applied Biosystems, Thermo Fisher Scientific). The housekeeping gene 18S was used to normalize fold change of each gene as compared to mock-infected control at the same time point and was calculated as ΔΔCT.

### 2.8. Quantification of MMP-3 and MMP-9

Serum concentration of total MMP-9 was measured at 2, 4 and 5 d.p.i. with VACV-WR, VACV-Wyeth, and VACV-Lister. Serum concentration of total MMP-3 was measured at 2, 4, and 5 d.p.i. with VACV-WR and VACV-Wyeth. For the quantification, Quantikine mouse MMP-9 or MMP-3 Immunoassay kit was used (R&D Systems Inc., Minneapolis, MN, USA) according to the manufacturer’s instruction. 

### 2.9. Examination of BBB Integrity by Evans Blue

Evans Blue (EB) dye (1% *w*/*v* in water for injection) was injected i.p. (0.8 mL/mouse). Mice were anesthetized 1 h later, perfused with PBS via intracardiac puncture until transparent drainage was observed. Brains were subsequently excised and photographed. 

### 2.10. Statistical Analysis

Two-tailed, unpaired Student’s *t*-test was used for comparisons between groups regarding the viral loads, ΔΔCT values in the early (2 d.p.i.) and late (4 and 5 d.p.i) comparisons and MMP-9 sera levels. A 2-way ANOVA was used to examine the signal intensity in the bioluminescence imaging for the effects of the viral strain, the time, and the interactions. Data were further analyzed by post-hoc *t*-tests. In all cases *p* < 0.05 was considered a significant difference. Statistics were performed using GraphPad Prism 5 for Windows (version 5.00, GraphPad Software Inc., San Diego, CA, USA).

## 3. Results

### 3.1. Disease Progression

**Figure 1:**
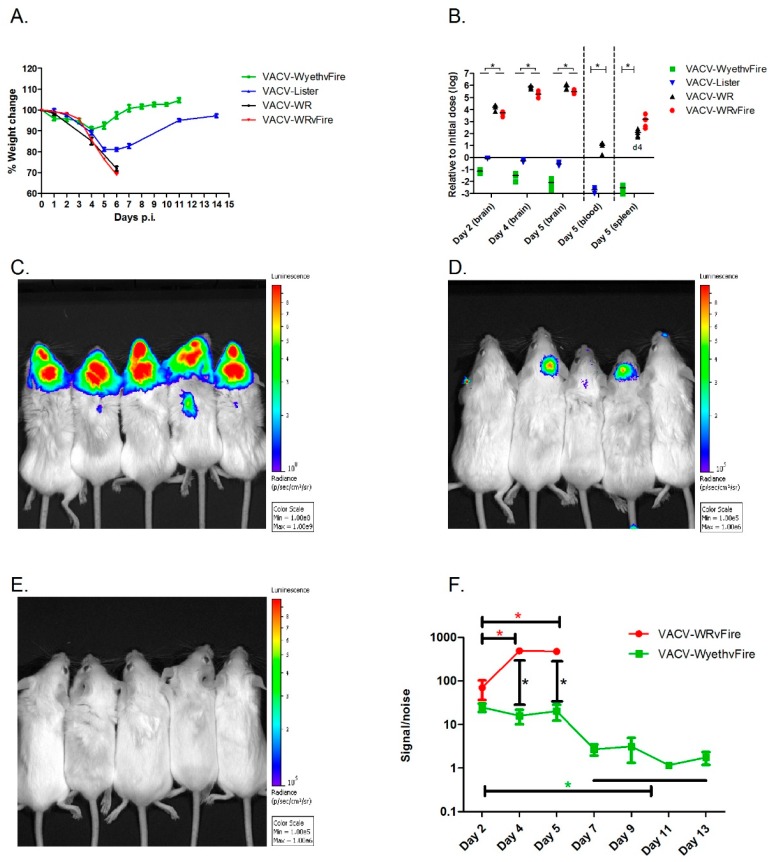
Disease progression following intracranial infection. Mice were injected intracranially with vaccinia virus-western Reserve (VACV-WR) (10^2^ pfu), VACV-WR with the vFire cassette (VACV-WRvFire) (10^2^ pfu), VACV-Lister (10^6^ pfu) or VACV-WyethvFire (10^6^ pfu). (**A**) Weight change following infection. (**B**) Viral loads, relative (log scale) to the initial infection dose, in brain (2, 4, 5 d.p.i.), blood (5 d.p.i.) and spleen (4 d.p.i. for VACV-WR, 5 d.p.i. for VACV-WRvFire and VACV-WyethvFire). All tissues were weight normalized for analysis. Asterisks denote *p* < 0.05 (*t*-test). (**C**) In-vivo whole body imaging following VACV-WRvFire at 5 d.p.i. (photon flux color scale: 10^8^–10^9^ p/s/cm^2^/sr). (**D**) In-vivo whole body imaging following VACV-WyethvFire at 5 d.p.i. (photon flux color scale: 10^5^–10^6^ p/s/cm^2^/sr). (**E**) In-vivo whole body imaging following VACV-WyethvFire at 7 d.p.i. (photon flux color scale: 10^5^–10^6^ p/s/cm^2^/sr). (**F**) Head signal intensity over the background noise at 2–5 d.p.i in VACV-WRvFire and 2–13 d.p.i in VACV-WyethvFire. Asterisks denote *p* < 0.05 in post-hoc *t*-test following 2-way ANOVA. Error bars represent standard errors.

To elaborate on the differences between lethal and non-lethal poxvirus infection of the CNS, BALB/c mice were infected intracranially (i.c.) with each of the vaccine strains: VACV-Lister, or VACV-WyethvFire, a derivative of the American NYBCH/Wyeth vaccine strain, modified to express the Firefly Luciferase reporter gene and Green Fluorescent Protein (GFP) (vFire cassette). In comparison, mice were similarly infected with VACV-WR, or VACV-WRvFire (VACV-WR with the vFire cassette). Brain infection with each of the vaccine strains at a dose of 10^2^ pfu was tolerated and neither morbidity nor mortalities were observed (not shown). Infection with much higher viral doses (up to 10^6^ pfu) of either VACV-Lister and or VACV-WyethvFire did not cause mortality and the observed signs of morbidity (transient weight loss and ruffled fur) that peaked at 4 to 5 d.p.i., were followed by full recovery within 8 to 11 d.p.i. (Figure 1A). In contrast, following infection with 10^2^ pfu VACV-WR all mice deteriorated rapidly and succumbed to the infection within 6 to 7 days (Figure 1A). Beside the observed weight loss, all VACV-WR infected mice had ruffled fur with hunched back posture and 30% also suffered from loss of balance and orientation at advanced stages of the disease. No differences were observed between the virulence of VACV-WR and that of VACV-WRvFire at the indicated viral doses and route of infection, suggesting that the vFire cassette does not affect the virulence of the virus at the tested conditions.

To find out whether the attenuated vaccine strains are capable of infecting the mouse brain but unable to further replicate there, mice were similarly infected with either 10^2^ pfu of VACV-WR or VACV-WRvFire or 10^6^ pfu of each of the vaccine strains and analyzed for viable viral content in the brain (Figure 1B). Within 48 h, the viral load of VACV-WR and VACV-WRvFire increased by four orders of magnitude compared to the initial infection dose, reaching a six logs increase by day five p.i. At this time point, VACV-WR spread from the brain and was detected in the blood and spleen (Figure 1B). In contrast, in VACV-Lister and VACV-WyethvFire infected animals, the virus was gradually cleared from the brain, and viral loads were significantly lower than VACV-WR infected brains. Additionally, no virus was detected in the blood or spleen (Figure 1B). To further substantiate the above data that VACV-WR efficiently replicates in the brain and spreads to peripheral tissues while the vaccine strains infect the brain and cleared, we used Luciferase-based whole body imaging following infection with either VACV-WRvFire (10^2^ pfu) or VACV-WyethvFire (10^6^ pfu). A 2-way ANOVA analysis showed a significant effect of the viral strain (F (1,24) = 177.6, *p* < 0.0001), the days (F (2,24) = 30.93, *p* < 0.0001) and the interactions between them (F (2,24) = 33.04, *p* < 0.0001). On day four and five p.i. the luminescence intensity following VACV-WRvFire infection was significantly higher than the intensity following VACV-WyethvFire infection (Figure 1C,D,F). On day five, the signal to noise ratio (S/N) following VACV-WRvFire infection was 476.9 ± 102.0 while the S/N following VACV-WyethvFire infection was significantly lower (20.4 ± 16.2; *n* = 5/group; P = 0.0007 (post-hoc *t*-test); Figure 1C,D,F). While a significant signal was observed from mice infected with VACV-WRvFire on a scale of 10^8^ to 10^9^ radiance (p/s/cm^2^/sr; Figure 1C), no signal was detected on the same scale in VACV-WyethvFire infected mice (data not shown). Only when the sensitivity was increased by 3 logs to a scale of 10^5^ to 10^6^, luminescence was detected in three out of five infected mice while no signal was observed in the rest of animals, indicating that the vaccines are cleared from the brain. On day five d.p.i., VACV-WRvFire was also detected in the periphery (Figure 1C) in agreement with the viral load data (Figure 1B). Seven d.p.i., luminescence was no longer detected in VACV-WyethvFire infected mice (Figure 1E,F). Overall, the data so far clearly demonstrates that while VACV-WR rapidly replicates in the brain and later in peripheral tissues, both VACV-Lister and VACV-WyethvFire (referred from this point as VACV-Wyeth) are capable of infecting the brain, but the viral load declines rapidly while remaining contained in the infection site.

### 3.2. Meninges and Lateral Ventricles Are the Main Sites of Interaction at Advanced Stages of Infection

**Figure 2:**
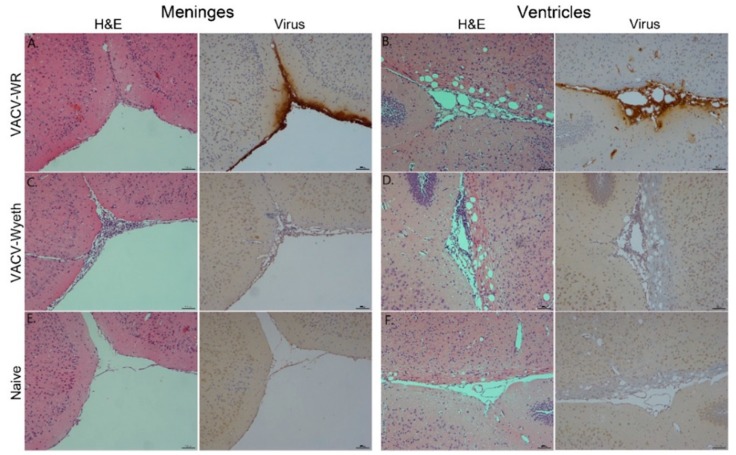
Detection of VACV-WR but not VACV-Wyeth antigens in the meninges and the ventricular system. Brain sections, 5 d.p.i with VACV-WR (10^2^ pfu) or VACV-Wyeth (10^6^ pfu) stained with Hematoxylin and Eosin (H&E) or with antibodies to VACV (positive stain in brown). (**A**,**B**) VACV-WR. (**C**,**D**) VACV-Wyeth. (**E**,**F**) Carrier-injected control. Meninges (A,C,E—serial sections) and ventricles (B,D,F—serial sections) areas are shown.

**Figure 3:**
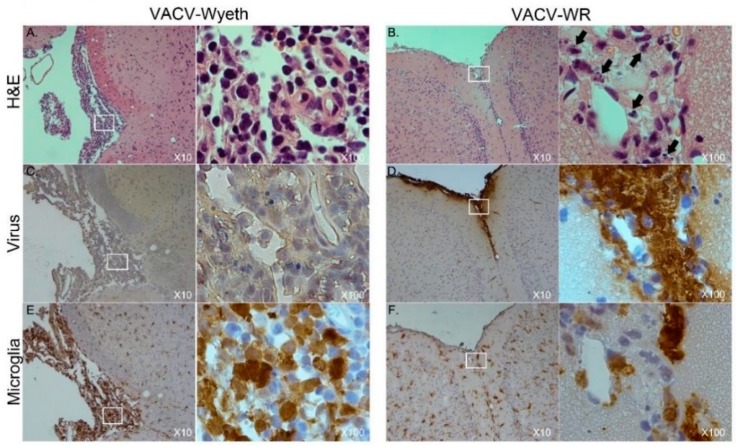
Macrophages/Microglia and virus-infected cells are almost mutually exclusive. Serial sections of meninges, 5 d.p.i with VACV-Wyeth (10^6^ pfu infection dose; A,C,E) or VACV-WR (10^2^ pfu infection dose; B,D,F). Brain sections were stained with H&E (**A**,**B**), antibodies to VACV (**C**,**D**) or antibodies to iba-1 antigen of macrophages / microglia cells (**E**,**F**); positive stain in brown. White box area enlarged (×100) on the right of each ×10 picture. Cell debris are marked with arrows in panel B (×100 enlargement).

To further elucidate on the spatial distribution of infected tissue, virus-infected cells, affected cell types, and immune cell infiltration we examined tissue sections from VACV-WR and VACV-Wyeth by histology at 5 d.p.i. While intense staining of viral antigens was detected in the brains of VACV-WR infected mice, the brains of VACV-Wyeth or VACV-Lister infected animals lacked any viral staining except for sporadic staining in some of the examined brains (Figure 2A–D). These results are in agreement with the data demonstrating propagation of VACV-WR but not VACV-Wyeth or VACV-Lister in mice brain (Figure 1B–E). VACV-WR propagation was mainly detected around the meninges, blood capillaries, and the ventricular system (Figure 2A,B; virus stain; Table 1). A moderate to high influx of mononuclear cells was detected in the VACV-Wyeth-infected brains in comparison to VACV-WR infection and naïve non-infected control, mainly around the meninges and ventricles (Figure 2E,H; Table 1). This infiltration was dominated by lymphocytes and macrophages/microglia (Figure 3A,E; Table 1). In contrast, in VACV-WR-infected brains, the number of immune cells in the meninges was significantly lower and was predominated by macrophages. Lymphocytes were found at low numbers (Figure 3B; Table 1) and cellular debris indicating of damaged cells, co-localized with viral antigens immunostaining (Figure 3B,D,F). 

Overall, histology analysis at 5 d.p.i. revealed that at this stage of disease, the meninges and the brain vasculature are the major sites of viral infection. In sharp contrast, viral clearance from these sites appears to correlate with robust infiltration of mononuclear cells, at significantly higher numbers—a phenomenon which was not apparent in VACV-WR infected brains. 

### 3.3. Vasculature Leakage Coincide with VACV-WR Dissemination from the Brain

**Figure 4:**
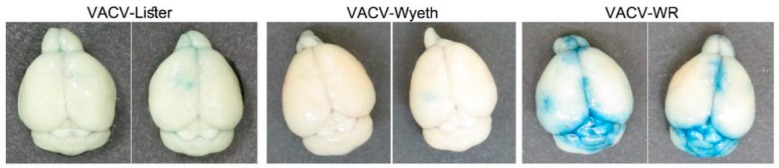
Infection with VACV-WR results in BBB breakdown and elevated levels of MMP-9. Mice were infected i.c. with VACV-Lister (10^6^ pfu, *n* = 4), VACV-Wyeth (10^6^ pfu, *n* = 5) or of VACV-WR (10^2^ pfu, *n* = 5). Perfused brains of VACV-Lister (left; 6 d.p.i), VACV-Wyeth (middle; 5 d.p.i.) and VACV-WR (right; 5 d.p.i.) following Evans-blue peripheral administration (two representative brains from each strain). Blue color represents vasculature dysfunctional leakage, a hallmark of BBB breakdown.

As previously shown, VACV-WR continues to propagate in the mice brains, and at advanced stages of the disease, namely at 5 d.p.i., VACV-WR was also detected in the blood and the spleen of the mice while VACV-Lister or VACV-Wyeth were not detected (Figure 1B,C). This finding suggests that at advanced stages of the disease, VACV-WR breaches out from the site of infection to the periphery. To elaborate whether this propagation was accompanied with blood–brain barrier (BBB) disruption, infected mice were injected peripherally with Evans-blue dye and monitored for blue staining in the perfused brain, as a measure of BBB breakdown. Indeed, BBB breakdown was detected in the brains of VACV-WR infected mice but not of VACV-Wyeth or VACV-Lister-infected animals indicating dysfunctional BBB in the VACV-WR infected brain tissue (Figure 4).

Overall, it appears that while clearance of the vaccine strains from the brain is associated with activation of immune cells and intact BBB, infection with the virulent VACV-WR strain is associated at the symptomatic stages of the disease with massive viral replication in the meninges and ventricles leading to tissue damage, BBB breakdown, and virus spread to the periphery.

### 3.4. Differential Brain Gene Expression Following Infection with Attenuated and Neurovirulent Viruses

**Figure 5:**
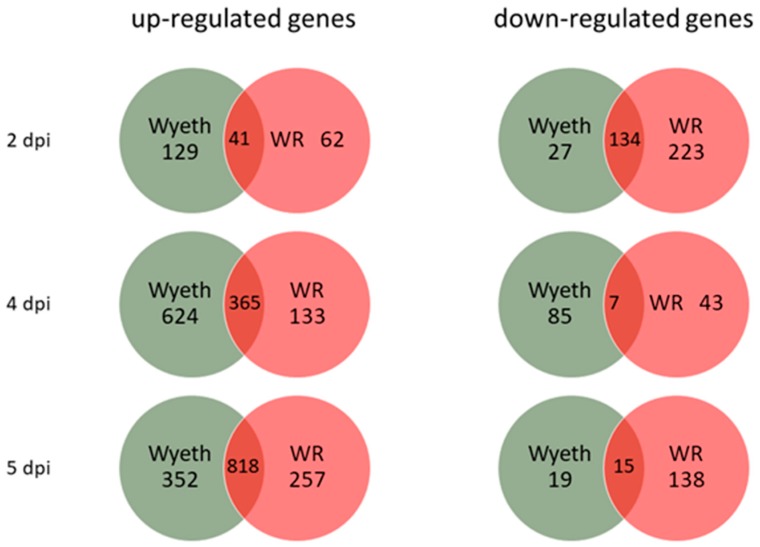
Quantification of up- and down-regulated genes following i.c. infection. Mice were infected with VACV-Wyeth and VACV-WR and total RNA-seq was done from brains 2, 4, and 5 d.p.i. Van diagrams representing number of genes that were significantly up- or down-regulated compared to the carrier control (*p* < 0.05, Fold change >2.0).

To study the differential response of the host brain tissue to infection with virulent and attenuated vaccine strains, RNA samples were prepared from whole brains 2, 4, and 5 d.p.i. and complete gene expression profile was compared utilizing whole transcriptome sequencing (RNA-seq). This schedule enabled us to examine the progression of the response to each virus for both early (asymptomatic) and late (symptomatic) stages of the disease (Figure 1A). Each group (3 animals/group/time point) was compared to the carrier, PBS + 2% FCS (PBF), injected control group at the corresponding time points to exclude any effects of the i.c. injection procedure. A cut off of >2.0 in fold change and *p* < 0.05 was used initially to examine the overall number of genes that were up- or down-regulated following the infection of each strain (Figure 5). Whereas VACV-Wyeth infection was associated with most up-regulated genes 2 and 4 d.p.i., on day 5, when mice were already morbid, most upregulated genes were commonly induced by both viruses. In contrast, VACV-WR infected mice had remarkably more down-regulated genes than VACV-Wyeth, on both early (2 d.p.i.) and late (5 d.p.i.) stages of the disease. Furthermore, and in contrast to the up-regulated gene analysis, more shared genes that were down-regulated were detected on day two than later days p.i.

In-depth analysis of the genes in each group indicated that the differences between the two groups originated mainly from genes related to different stages in the immune and inflammatory responses. In general, the gene expression profile in the VACV-Wyeth infected groups pointed to robust and rapid immune response which preceded that of VACV-WR, while at the late stages of the infection, genes related to tissue damage were robustly upregulated in the VACV-WR. At 2 d.p.i. with VACV-Wyeth, within the asymptomatic incubation period, several genes related to innate immunity and antiviral response were significantly upregulated compared to the VACV-WR. These included interferon-related genes, such as *Ifi44, Irf7, Ifi27l2a, Ifit1*, and *Ifit3*, two members of the Schlafen (Slfn) family, *Slfn1* and *Slfn4*, the ubiquitin-like modifier *ISG15* and Z-DNA binding protein 1 (*Zbp1*) (Table 2). All the above genes, which play a role in the induction of innate response and anti-viral responses, which were significantly induced in brains of the VACV-Wyeth infected mice, remained almost unaffected following VACV-WR infection (Table 2).

At 4 d.p.i., brain gene expression profiles differed significantly between the two groups. Brain infection with VACV-Wyeth resulted in robust induction of immune response related genes including antigen presentation and recruitment and activation of immune cells (Table 3; fold change (*p* values are presented in Supplementary Table S2)). This list included genes related to the complement system (*C1ra; C1rb; C1s; C2; C3; C6*), Major Histocompatibility Complex (MHC) class I and II (*H2-Ha; H2-Eb1; H2-K1*) and different cluster of differentiation receptors (*CD3d, CD3e, CD3g; CD8a, CD8b1; CD2; CD27; CD5*). Various chemokine genes, responsible for the control and recruitment of immune cells to the site of infection (i.e., *Ccl2, Ccl4, Ccl5, Ccl7, Ccl8, Ccl12,* and *Ccl19*), were also significantly upregulated in the VACV-Wyeth infected brains on 4 d.p.i (Table 3). In addition, microglia related genes, and genes associated with microglial cell activation (*Mki67, Cd40, Tmem119* and *Cx3cr1*) were significantly induced 4 days following VACV-Wyeth infection indicating the possible role of these cells in the induction of immune response in the brain. In sharp contrast, the same genes listed above were poorly induced, if at all, following infection with VACV-WR. For example, chemokine genes, such as *Ccl2*, 5, 7, and 12, were induced following infection with VACV-WR, yet to a lesser extent than following VACV-Wyeth infection (Table 3).

Compared to 4 d.p.i, analysis of gene expression 5 d.p.i. with VACV-Wyeth reveals that expression level of most of the genes remained high, except for two sets of genes that were further induced including the CD3 and CD8 gene families and T-cell activation genes, such as *Prf1*, *Gzma* and *Gzmb* (×1.5 fold increase, average of all the VACV-Wyeth genes in Table 3). Interestingly, these genes were also induced in VACV-WR infected brains 5 d.p.i but to an overall lesser extent than in VACV-Wyeth infected brains. While expression of chemokines, such as Ccl2, Ccl4, Ccl7 and *Ccl12*, was induced between 4 and 5 d.p.i. in VACV-WR infected brains, their expression in VACV-Wyeth infected brains was reduced. Perforin and granzyme genes that were induced by the vaccine strain already on 4 d.p.i. were significantly induced only on 5 d.p.i. following infection with VACV-WR (×7.5 fold increase, average of all the genes in Table 3 for VACV-WR).

Interestingly, expression of genes encoding matrix metalloproteinase (MMP) -3, 8, 13, and 19 and of tissue inhibitor of metalloproteinase 1 (Timp1) was significantly higher in VACV-WR infected brains than in VACV-Wyeth infected brains (mainly on 5 d.p.i.). Various MMPs and their regulators play an important role in tissue integrity. In different pathologies, the levels of these genes are significantly induced.

Altogether, the gene expression profile following infection with the vaccine strain indicates that induction of innate and adaptive immune response in the brain is rapid and robust, but significantly delayed and weak following infection with the neurovirulent strain. However, genes associated with tissue damage, such as MMPs, were strongly induced at late stages of the disease in VACV-WR infected brains and to a lesser extent following the vaccine strain infection. 

### 3.5. Pathway Analysis of Gene Expression Profiles

To further elaborate on the cellular pathways in the brain that took place following infection with VACV-Wyeth and VACV-WR, we applied ingenuity pathway analysis (IPA) to the above data. A threshold of *p* < 0.05 and a fold change higher than ±30% compared to the PBF control were used, and 1500 to 2500 genes which passed this threshold in each comparison served for the analysis. Overall, the top activated canonical pathways associated with VACV-Wyeth infection were related to the brain immune response while in the VACV-WR brains the top pathways were related to damage and acute responses (Supplementary Figure S1).

**Figure 6:**
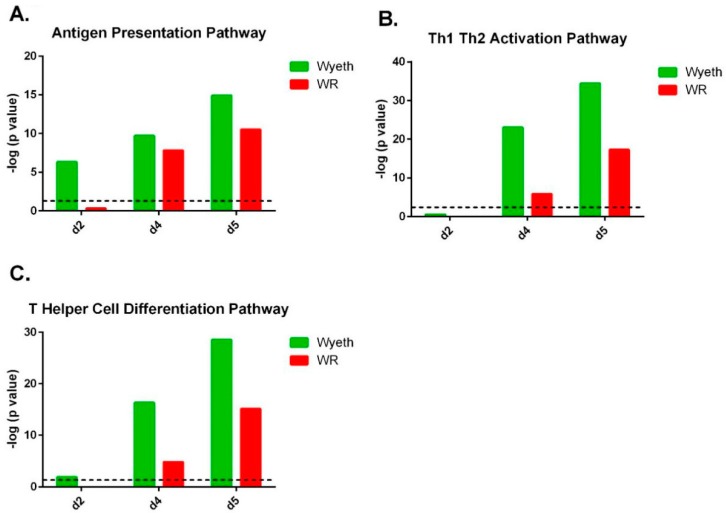
Clearance of VACV-Wyeth from infected brain is associated with robust induction of brain immunity pathways. Ingenuity pathway analysis (IPA) analysis based on brain gene expression of i.c. infected mice with 10^6^ pfu of VACV-Wyeth or 10^2^ pfu of VACV-WR at 2, 4, and 5 d.p.i. (**A**) Antigen Presentation Pathway. (**B**) Th1-Th2 Activation Pathway. (**C**) T Helper Cell Differentiation Pathway. Dashed line marks *p* value of 0.05.

Pathway analysis of the gene expression profile following VACV-Wyeth infection revealed robust activation of the antigen presentation pathway which scored a −log (*p* value) of 6.3 on day two p.i. increasing to even more significant values on day four (9.7) and five (14.9; Figure 6A; Supplementary Figure S1 (canonical pathway #17); Supplementary Figure S2). At this relatively early time point (2 d.p.i.), in which the viral burden of VACV-WR was comparable or slightly higher than in VACV-Wyeth (5.1 × 10^5^ for VACV-WR (*n* = 5; range 2.5 × 10^5^–6.7 × 10^5^) and 7.4 × 10^4^ pfu/brain for VACV-Wyeth (*n* = 5; range 4.9 × 10^4^–1.0 × 10^5^), Figure 1B), induction of the antigen presentation pathway related genes was not observed in VACV-WR infected brains (−log (*p* value) of 0.3 on 2 d.p.i.; Figure 6A; Supplementary Figure S1 (canonical pathway #26); Supplementary Figure S2). Additionally, following VACV-Wyeth infection, we observed robust activation of Th1, Th2 and T Helper cell differentiation pathways reaching high −log (*p* values) of 34.4 and 28.5 on 5 d.p.i. (Figure 6B,C; Supplementary Figure S1 (canonical pathway #1, 3 in the VACV-Wyeth-infection and #12, #21 in the VACV-WR-infection, respectively)).

The top canonical pathways in the VACV-WR infected brains better correlated with mechanisms of tissue damage and acute responses while pathways associated with induction of immune response were poorly activated and if so, were delayed and weak compared to VACV-Wyeth infected brains (Supplementary Figure S1). In addition, the IPA analysis revealed stronger activation of stress response pathways in VACV-WR compared to VACV-Wyeth infected brains, at an early time point (2 d.p.i.). These pathways included mitochondrial dysfunction (VACV-Wyeth −log (*p* value) of 6.6; VACV-WR −log (*p* value) of 13.7) and Oxidative Phosphorylation (VACV-Wyeth −log (*p* value) of 7.0; VACV-WR −log (*p* value) of 14.2; Supplementary Figures S3 and S4, respectively).

The efficient and robust activation of immune response following infection with VACV-Wyeth, continued in later days and on four to five d.p.i., induction of related pathways as CD28 in T Helper Cells, Communication between innate and adaptive immune cells and T cell receptor signaling were observed (Supplementary Figure S1, VACV-Wyeth pathways # 15, 21, 26, respectively). In contrast, following infection with VACV-WR, the communication between innate and adaptive immune cells pathway was ranked on the 33rd place while the other two were not in the top 34 places. 

Overall, analysis of the gene expression profiles following infection with the attenuated, vaccine strain VACV-Wyeth and the neurovirulent strain VACV-WR, revealed that clearance of VACV-Wyeth was accompanied by robust local innate and adaptive immune response related genes and pathways whereas the immune response to the virulent VACV-WR was inferior and delayed, concomitantly with strong induction of tissue damage and stress responses related genes.

### 3.6. Verification of the Gene Expression Analysis

**Figure 7:**
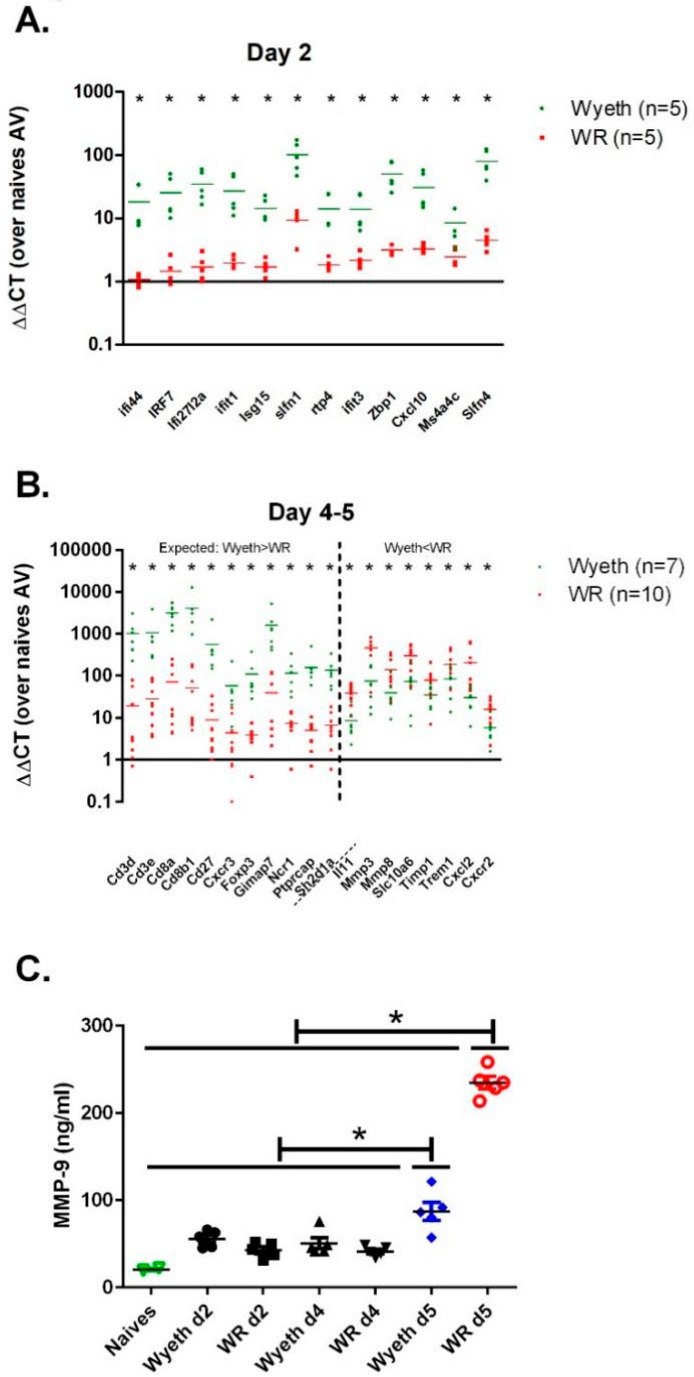
Verification of gene expression analysis. Expression of selected genes was measured by quantitative real-time RT-PCR on brain RNA isolated from mice infected i.c. with VACV-Wyeth (10^6^ pfu) or VACV-WR (10^2^ pfu) 2 (**A**) 4 or 5 d.p.i (**B**). Vertical dashed line in panel B, marks the border between the set of genes that were expected to be higher in the VACV-Wyeth compared to the VACV-WR, based on the whole genome RNA seq (left to the line) and the genes that were expected to be higher in the VACV-WR compared to VACV-Wyeth (right to the line). (**C**) MMP-9 protein levels in sera of naive mice, VACV-Wyeth and VACV-WR infected mice at 2, 4, and 5 d.p.i. Asterisks denote *p* < 0.05 (*t*-test).

The RNA-seq of brain samples from the early and late stages of infection (day two, days four to five p.i., respectively) highlighted genes with expression levels that differed significantly between the VACV-Wyeth and the VACV-WR infected mice. To verify the solidity of the RNA-seq results we selected 31 genes that were differentially expressed following infection with VACV-Wyeth or VACV-WR and measured their expression in RNA samples from infected mice utilizing quantitative real-time RT-PCR. To increase the sample size and improve the statistical value of the results, we added additional samples to the samples already analyzed by the RNA-seq. In the selected early-induced host genes, we evaluated genes which were highly expressed in the VACV-Wyeth-infected brains compared to VACV-WR-infection. At the late genes group, we evaluated the expression of genes that were either higher in VACV-Wyeth infection compared to the VACV-WR infection or lower. Altogether, data of the RT-PCR verification confirmed the RNA-seq data. During the early asymptomatic period (2 d.p.i.) 12 genes were highly expressed following VACV-Wyeth and poorly following VACV-WR. Their elevated expression correlated with good prognosis (Figure 7A). At the late—symptomatic stage of the disease (days four to five p.i.) in which the mice infected with either of the strains showed signs of morbidity (i.e., weight loss and ruffled fur, Figure 1A), a set of 11 genes were significantly upregulated in the VACV-Wyeth-infected group compared to the VACV-WR-infection while other eight genes were significantly elevated in the VACV-WR-infected brains as compared to the VACV-Wyeth-infection (Figure 7B). 

In contrast to the induced immune response following VACV-Wyeth infection, the outcome of VACV-WR infection was more indicative of tissue damage. To further elaborate on the gene expression profile and tissue damage at the protein level, we determined the serum levels of MMP-9 and MMP-3 at several days p.i. At 5 d.p.i. the serum levels of MMP-9 in the VACV-WR infected mice were significantly higher than on earlier days or following infection with the vaccine strains (Figure 7C, VACV-Lister). Serum MMP-3 levels were not elevated. Overall, elevation of serum MMP-9 at advanced stages of the disease correlated with BBB breakdown (Figure 4) and disease severity in VACV-WR infected mice. 

## 4. Discussion

In the current study, we aimed to elaborate on the viral and host interplay during poxvirus infection of the CNS. We asked: what are the mechanisms underlying the control of viral replication in the brain (e.g., in attenuated strains) and what allows for virus replication, brain pathology and virus dissemination as observed following infection with virulent viruses (like VACV-WR)? We infected the mice intracranially aiming to focus on the virus-brain interactions and to exclude differences between the viruses in the response to viral entry and dissemination from the peripheral tissues. 

Our data indicate that brain infection of immune competent mice with attenuated strains induces robust antiviral immune response leading to viral clearance and recovery. On the contrary, infection with VACV-WR leads to weak and aberrant immune response and concomitant induction of tissue damage pathways associated with robust viral replication in the brain, dissemination to the periphery and lethal outcome.

Gene expression analysis revealed that following VACV-Wyeth brain infection, several host genes were induced including genes involved in the induction of innate and adaptive immune response, while these genes were not induced, or induced to a significantly lesser extent following infection with VACV-WR. The reduced expression level of immune stimulating genes may be attributed to inefficient viral replication. However, we show here that VACV-WR replicates efficiently in the mouse brain (Figure 1, Figure 2 and Figure 3), suggesting an active immune modulation by VACV-WR. The results of the gene expression analysis and IPA clearly show that induction of antiviral immunity is severely inhibited including antigen presentation, Th1 and Th2 activation and interferon-mediated signaling pathways (Supplementary Figure S1). Unlike other organs, robust antiviral activity in the brain might have severe consequences since the brain has only limited capacity to maintain functionality or to regenerate following cytotoxic damage to infected cells. Indeed, upon infection with the vaccine strains, rapid and robust immune response prevent excessive viremia and damage, and as late as 5 d.p.i., the immune response in the brain is controlled, concomitantly with viral clearance. Upon infection with the virulent strain VACV-WR, the immune response is weak at early stages of the disease, induced late, uncontrolled, and is accompanied by induction of stress response and genes associated with tissue damage (e.g., MMPs) and the consequence is brain damage and death. Mechanisms of immune evasion associated with VACV infection include, among others, neutralization of complement factors, cytokines, chemokines, and interferons secretion [26,27]. Induction of glucocorticoid receptor signaling pathway in the brain followed VACV-WR infection but not VACV-Wyeth indicating a possible role for this pathway in pathogenesis induced by VACV-WR (Supplementary Figure S1). The role of steroid hormone in virulence of vaccinia virus has been previously described [28]. Following infection with the vaccine strain we observed differential expression of genes related to various components of the immune response including interferon-related genes, antigen presentation, recruitment and activation of immune cells, complement system, MHC class I and II, different cluster of differentiation receptors (as CD3, CD8), and genes associated with microglial cell activation (Table 2 and Table 3). These genes were poorly and lately induced following infection with the neurovirulent strain VACV-WR. 

Using multiple analyses including plaque formation, immunohistochemistry, and bioluminescence-based whole body imaging we demonstrate that the vaccine strain transiently replicates in the brain but is rapidly cleared and does not spread to other organs. Our unpublished data, using immune-deficient mice, indicate that it is mainly the immune response rather than the replication capacity of the vaccine strain that controls the replication in the mouse brain. Whether the neurovirulence capacity of VACV-WR involves not only the ability to attenuate the immune response in the brain as shown here but also additional, non-immune, virulence mechanisms, mainly within the brain, remains elusive. Intracranial infection of mice with poxviruses has been previously used to evaluate the importance of various proteins (i.e., N1L [17]), pathogenesis of different orthopoxviruses [20], and to characterize the cellular immune response following sublethal infection [18]. Viral proteins which contribute to the virulence of VACV-WR through modulation of the host immune response include C4, N2, B13, B14, 169, C6, N1, and F1 [29,30,31,32,33,34,35]. However, in the context of neurovirulence, their contribution to the virulence of VACV-WR through modulation of the immune response was not fully addressed. 

Immunohistochemical analyses of the brain sections revealed that at late stages of the infection with the virulent strain VACV-WR, most of the viral antigens are localized within the brain in the meninges, the ventricles and blood vessels. The mechanisms underlying this distribution pattern are not clear. Yet, these regions are located at the border between the brain parenchymal tissue and the external tissues better defining the disease as meningoencephalitis. Various models of virus infection of the brain use neonatal mice (p3) as for many viruses including alpha and flavi- viruses, age plays a critical role in disease progression [36,37]. It was previously shown that VACV infects astrocytes in the neonatal brain [21]. Whether maturation of the immune system allows not only the development of robust and protective immune response but also restricts the susceptibility of various brain cell types, such as astrocytes and microglia, to infection, awaits further study. In addition, the localization of VACV-WR at this late stage of the disease, the BBB breakdown, and the virus dissemination to peripheral organs, including the blood and spleen, may indicate the shortage of replication resources and exhaustion of virus-susceptible brain tissues. Previous study elaborated on the role of endothelial cells in VACV-WR spreading into the brain and their involvement in BBB damage and brain pathology [38]. It is interesting to note that *Gkn3* gene was the utmost downregulated gene in VACV-WR infected brains at day five p.i. (22.6 fold change, *p* value: 2 × 10^−20^). This gene is related to inhibition of gastric epithelial cells [39] and was also shown to be significantly downregulated in spleens of mice infected with *Toxoplasma gondii* [40] or West Nile virus [41]. Such significant downregulation of this gene combined with the significant upregulation of MMPs and the excessive BBB permeability might serve as an indicative marker of brain vasculature disorder at the advanced stages of the disease.

PVE is a severe adverse reaction [42,43,44]. To prevent it, vaccine stocks are routinely screened, and development of new vaccines require an evaluation of the encephalitic potential of the candidate vaccines (EMEA, 2002, Development of vaccinia virus-based vaccines against Smallpox, CPMP/1100/02). However, quantitative markers and established models and criteria are lacking. Analysis of the differential gene expression following brain infection with the virulent VACV-WR strain or the attenuated VACV-Wyeth strain revealed several host genes that differed significantly between pathogenic and protective responses in the brain and characterize encephalitis/meningitis infection. Although analysis of these genes in the brain might not be favorable or technically applicable as a clinical marker of meningoencephalitis, we suggest that these genes or pathways are potential prognostic markers. Indeed, based on the gene expression profile which demonstrated the robust induction of MMP genes following infection with VACV-WR, we measured the level of MMP-9 in the mouse serum of infected mice and showed that the level of serum MMP-9 protein increases significantly in late stages of the disease suggesting its potential as a surrogate marker of BBB damage. Interestingly, Ichiyama et al. showed that high MMP-9 sera levels were detected in patients suffering from measles virus related subacute sclerosing panencephalitis brain inflammation [45,46]. MMP-3 mRNA was significantly induced in the brain following VACV-WR infection. Interestingly, MMP-3 was similarly elevated in the brain during encephalitis induced by West Nile virus supporting its role in encephalitis [47]. Whether other gene products listed here can be applied for non-invasive analysis of viral infection of the brain is yet to be discovered. In the analysis shown here, we distinguish between early markers of encephalitis (pre-symptomatic) and markers with late-onset (symptomatic). This definition has an important role when addressing the prevention of brain damage before symptoms arise. For that purpose, markers that can be measured non-invasively and in an unbiased way can be of high importance. We further suggest that the differentially expressed genes uncovered in this study can potentially be applied in the future for analyses of vaccine efficacy, as the analysis uncovered "foot-prints" of genes that highly correlate with induction of rapid, robust, and protective immunity, mediated by brain and systemic effectors. We speculate that this unique and significantly efficacious immune response might be highly valuable when addressing needs or correlates for rapid, robust and protective immunity. 

In this work, we compared the response to brain infection with a neurovirulent strain compared to attenuated vaccine strains. Multiple analyses indicated that robust immune response in the brain is associated with recovery while delayed and uncontrolled immune response and robust induction of tissue damage following infection with the neurovirulent strain is associated with encephalomyelitic disease followed by virus dissemination and lethal outcome. This work highlights multiple pathways and genes associated with brain immunity and damage that might be valuable in the clinic as prognostic markers and in the evaluation and design of novel therapeutics and vaccines.

## 5. Conclusions

In this work, we examined the response to brain infection with attenuated vaccinia vaccine strains compared to a neurovirulent strain. Multiple analyses indicated that following attenuated viral infection, a robust immune response in the brain, was associated with viral clearance and recovery. On the contrary, delayed and uncontrolled immune response combined with robust induction of tissue damage were detected following infection with the neurovirulent strain. This type of infection was associated with meningo-encephalomyelitic disease followed by viral dissemination and lethal outcome. This work highlights multiple pathways and genes associated with brain immunity and damage. Following attenuated strains infection, a robust immune response combined of antigen presentation, recruitment, Th1 Th2 activation and T cell differentiation was observed while in the neurovirulent strain the response was modulated, delayed and associated with genes and pathways indicating on damage. This information might be valuable in the clinic as prognostic markers and in the evaluation and design of novel therapeutics and vaccines.

## Figures and Tables

**Table 1:** Histology evaluation of viral presence and immune response at the symptomatic stage following infection.

Group	Virus		Inflammation Score	White Blood Cells (%)			
	Meninges Cortex	Lateral Ventricles		Lymphocytes	Macrophages	Neutrophil	Eosinophil
VACV-Wyeth	0	2	3	70	20	10	0
	0	1	3	75	20	5	0
	0	0	3	70	20	10	0
VACV-WR	3	3	1	10	70	20	0
	3	3	1	5	70	25	0
	3	2	1	5	70	25	0
Naives	0	0	0	0	0	0	0

Grades for viral presence and inflammation score (semi-quantitative): 0 = No positive cells; 1 = Very few positive cells (<10 cells/field); 2 = Few positive cells/Mild inflammation (>10 and <20 cells/field); 3 = Moderate number of positive cells/Moderate inflammation (>20 and <50 cells/field); 4 = High number of positive cells/Severe inflammation (>50 cells/field); Field: × 20 for viral stain; × 20 for inflammation score.

**Table 2:** Gene expression profile of induced innate immunity 2 days following VACV-Wyeth infection of the CNS.

Gene	VACV-Wyeth		VACV-WR				
	Fold Change ^	*p* Value	Fold Change ^	*p* Value		Fold Change	
*Ifi44*	9.8	9.8 × 10^−47^	−1.3	0.31			25
*Irf7*	14.0	5.8 × 10^−85^	1.6	0.01			
*Ifi27l2a*	17.9	1.6 × 10^−49^	2.2	2.2 × 10^−3^			
*Ifit1*	8.0	1.2 × 10^−60^	1.2	0.40			
*Ifit3*	7.4	7.9 × 10^−50^	1.2	0.34			
*Slfn1*	5.5	3.7 × 10^−7^	1.2	0.29			
*Slfn4*	12.6	1.7 × 10^−17^	1.6	0.18			
*Isg15*	13.2	6.2 × 10^−52^	2.6	6.5 ×10^−5^			0
*Zbp1*	13.0	1.9 × 10^−25^	1.8	0.07			−1

^ Color scale bar indicating the fold change magnitude appears on the right.

**Table 3:** Differential gene expression profile of immune response and tissue pathology 4 and 5 d.p.i. with VACV-Wyeth and VACV-WR.

Gene	Fold Change ^						
	Day 4 p.i.		Day 5 p.i.				
	Wyeth	WR	Wyeth	WR			
*C1ra*	15.2	3.0	12.1	9.7		**Fold change**	
*C1rb*	11.1	2.9	6.6	5.6			500
*C1s*	21.7	3.1	27.2	13.4			250
*C2*	7.2	1.8	9.4	5.4			
*C3*	22.7	3.4	26.6	18.5			
*C6*	7.4	1.2	3.6	2.5			
*H2-Aa*	23.5	2.2	48.0	12.6			
*H2-Eb1*	33.8	2.5	44.5	14.2			100
*H2-K1*	13.4	2.4	22.4	9.5			
*Cd3d*	32.0	1.5	76.7	7.4			
*Cd3e*	41.0	2.8	98.6	13.0			
*Cd3g*	28.3	1.5	99.5	8.5			
*Cd8a*	49.4	1.9	145.9	13.5			
*Cd8b1*	26.7	1.6	104.6	6.1			10
*Cd2*	25.6	-1.1	44.5	7.9			
*Cd27*	25.4	1.1	45.3	4.6			
*Cd5*	3.5	-1.6	9.1	1.3			0
*Ccl2*	88.7	14.7	27.0	103.5			
*Ccl4*	22.0	2.2	10.5	33.9			−2
*Ccl5*	93.0	9.0	120.2	21.3			
*Ccl7*	68.1	7.8	32.7	58.0			
*Ccl8*	25.7	2.6	50.3	11.7			
*Ccl12*	55.1	13.1	20.3	23.5			
*Ccl19*	8.0	−1.2	7.7	3.0			
*Mki67*	4.6	1.4	11.2	4.3			
*CD40*	5.0	−1.2	6.5	3.7			
*Tmem119*	1.9	1.0	1.9	−1.1			
*Cx3cr1*	1.1	−1.3	1.1	−2.0			
*Prf1*	42.4	1.1	53.8	40.6			
*Gzma*	138.3	6.5	203.6	71.8			
*Gzmb*	277.5	4.2	361.9	325.7			
*Mmp3*	4.2	22.4	1.4	34.4			
*Mmp8*	2.2	4.6	2.9	54.7			
*Mmp13*	1.4	3.7	2.3	7.0			
*Mmp19*	2.7	3.6	2.7	8.4			
*Timp1*	7.8	18.8	7.1	34.6			

^ Color scale bar indicating the fold change magnitude appears on the right.

## Supplementary Materials

The following are available online at https://www.mdpi.com/2076-393X/7/1/19/s1, Figure S1. Top canonical pathways following infection with VACV-Wyeth and VACV-WR. IPA analysis of brain gene expression 2, 4, and 5 d.p.i with VACV-Wyeth (106 pfu) and VACV-WR (100 pfu). Shared pathways are color coded respectively, Figure S2. Antigen Presentation Pathway. Genes associated with antigen presentation following infection with (A) VACV-Wyeth (left) and (B) VACV-WR (right) 2 d.p.i. Differentially expressed genes are highlighted in color. Color intensity indicates the degree of up-regulation (red) or down-regulation (green) relative to the day two mock infected brains. Solid lines represent direct interactions. -log(*p*-values) of the pathway are indicated. (For interpretation of the references to color in this figure legend, the reader is referred to the web version of this article), Figure S3. Mitochondrial Dysfunction Pathway. Genes associated with mitochondrial dysfunction following infection with (A) VACV-Wyeth (left) and (B) VACV-WR (right) 2 d.p.i., Figure S4: Oxidative Phosphorylation Pathway. Genes associated with oxidative phosphorylation following infection with (A) VACV-Wyeth (left) and (B) VACV-WR (right) 2 d.p.i. Differentially expressed genes are highlighted in color. Color intensity indicates the degree of up-regulation (red) or down-regulation (green) relative to the day two mock infected brains. Solid lines represent direct interactions and dashed lines represent indirect interactions. -log(*p*-values) of the pathway are indicated. (For interpretation of the references to color in this figure legend, the reader is referred to the web version of this article), Table S1. Selected early (2 d.p.i.) and late (4–5 d.p.i.) differentiating genes used for quantitative real-time RT-PCR, Table S2: Significant brain gene expression level following VACV infection (corresponding to Table 3).
