# Supplementary material for: Differential Response Following Infection of Mouse CNS with Virulent and Attenuated Vaccinia Virus Strains
Source: Vaccines (Basel). 2019 Feb 12;7(1):19. doi: 10.3390/vaccines7010019 (PMC6466266; doi:10.3390/vaccines7010019)
Supplement: Supplementary file 1 [file vaccines-07-00019-s001.pdf]

vaccines

MDPI

Article

Differential Response Following Infection of Mouse CNS with Virulent and Attenuated Vaccinia Virus Strains

Tomer Israely [1,†], Nir Paran [1,†], Noam Erez [1], Lilach Cherry [1], Hadas Tamir [1], Hagit Achdout [1], Boaz Politi [1], Ofir Israeli [2], Galia Zaide [2], Inbar Cohen-Gihon [2], Einat B. Vitner [1], Shlomo Lustig [1] and Sharon Melamed [1,*]

[1] Department of Infectious Diseases, Israel Institute of Biological Research (IIBR), Ness-Ziona, Israel; tomeri@iibr.gov.il (T.I.); nirp@iibr.gov.il (N.P.); noame@iibr.gov.il (N.E.); Lilachc@iibr.gov.il (L.C.); hadast@iibr.gov.il (H.T.); hagita@iibr.gov.il (H.A.); boazp@iibr.gov.il (B.P.); einatv@iibr.gov.il (E.B.V.); shlomolustig@gmail.com (S.L.)

[2] Department of Biochemistry and Molecular Genetics, Israel Institute of Biological Research (IIBR), Ness-Ziona, Israel; ofiri@iibr.gov.il (O.I.); galiaz@iibr.gov.il (G.Z.); inbarg@iibr.gov.il (I.C.-G.)

† Authors contributed equally to the article

* Correspondence: sharonm@iibr.gov.il; Tel.: +972-9-9381646

Supplementary Materials

Table S1. Selected early (2 d.p.i.) and late (4-5 d.p.i.) differentiating genes used for quantitative real-time RT-PCR.

Gene#	Early (2 d.p.i.)	Gene #	Late (4-5 d.p.i.)
1	Ifi44-Mm00505670_m1	1	Cd3d-Mm00442746_m1
2	Irf7-Mm00516793_g1	2	Cd3e-Mm01179194_m1
3	Ifi27l2a-Mm01329883_gH	3	Cd8a-Mm01182107_g1
4	Ifit1-Mm00515153_m1	4	Cd8b1-Mm00438116_m1
5	Isg15-Mm01705338_s1	5	Cd27-Mm01185212_g1
6	Slfn1-Mm00488306_m1	6	Cxcr3-Mm99999054_s1
7	Rtp4-Mm00469759_m1	7	Foxp3-Mm00475162_m1
8	Ifit3-Mm01704846_s1	8	Gimap7-Mm00525315_m1
9	Zbp1-Mm01247052_m1	9	Ncr1-Mm01337324_g1
10	Cxcl10-Mm00445235_m1	10	Ptprcap-Mm01236556_m1
11	Ms4a6c-Mm00459296_m1	11	Sh2d1a-Mm01316997_m1

12	Slfn4-Mm01298330_m1	12	Il11-Mm00434162_m1
13	18S-Hs99999901_s1	13	Mmp3-Mm00440295_m1
		14	Mmp8-Mm00439509_m1
		15	Slc10a6-Mm00512730_m1
		16	Timp1-Mm01341360_g1
		17	Trem1-Mm01278455_m1
		18	Cxcl2-Mm00436450_m1
		19	Cxcr2-Mm99999117_s1
		20	18S-Hs99999901_s1

Table S2. Significant brain gene expression level following VACV infection (correspomding to Table 3).

	p value			
Gene	Day 4 p.i.		Day 5 p.i.	
	Wyeth	WR	Wyeth	WR
C1ra	6.5E-58	6.6E-07	1.2E-19	1.1E-46
C1rb	1.1E-07	6.9E-03	3.3E-05	5.7E-05
C1s	1.6E-87	6.0E-07	6.6E-102	4.8E-65
C2	1.4E-51	2.3E-03	8.7E-13	1.8E-26
C3	7.6E-30	3.6E-09	5.4E-32	1.2E-139
C6	1.6E-05	4.7E-01	1.1E-02	7.4E-02
H2-Aa	6.7E-67	4.0E-04	6.6E-80	5.4E-65
H2-Eb1	3.3E-140	3.2E-03	5.1E-57	2.7E-61
H2-K1	2.1E-284	2.4E-14	1.7E-130	6.7E-199
Cd3d	2.7E-17	2.1E-01	1.3E-27	5.0E-05
Cd3e	2.9E-22	1.0E-02	4.8E-36	4.6E-09
Cd3g	4.1E-17	2.4E-01	3.4E-32	8.4E-06
Cd8a	4.6E-25	9.3E-02	1.1E-44	3.1E-09
Cd8b1	7.2E-14	1.6E-01	1.2E-40	1.1E-04
Cd2	1.4E-17	7.0E-01	1.3E-28	9.0E-07
Cd27	5.5E-16	8.0E-01	2.4E-22	1.7E-03
Cd5	9.6E-05	2.1E-01	9.8E-21	4.6E-01
Ccl2	3.3E-68	3.8E-21	5.1E-14	8.5E-80
Ccl4	3.0E-19	NA	5.6E-10	1.4E-32
Ccl5	3.6E-41	2.3E-09	4.7E-74	3.4E-23
Ccl7	4.1E-37	2.4E-08	2.2E-22	3.5E-42

Ccl8	9.5E-21	1.7E-02	3.0E-28	1.4E-09
Ccl12	1.2E-64	6.1E-19	1.2E-15	5.9E-18
Ccl19	2.7E-11	6.4E-01	1.8E-11	2.0E-03
Mki67	7.3E-27	9.0E-02	8.8E-83	6.1E-19
CD40	3.9E-11	6.1E-01	3.0E-12	9.7E-06
Tmem119	4.4E-22	8.7E-01	9.7E-17	5.2E-01
Cx3cr1	1.8E-01	1.1E-02	7.4E-02	5.6E-25
Prf1	5.2E-21	6.3E-01	6.92E-22	4.07E-19
Gzma	2.3E-42	2.2E-06	1.07E-45	8.17E-29
Gzmb	2.0E-55	3.6E-04	2.39E-48	1.25E-55
Mmp3	2.6E-03	1.1E-17	5.0E-01	8.7E-22
Mmp8	1.0E-01	1.3E-04	4.2E-02	1.3E-21
Mmp13	5.0E-01	8.2E-04	1.0E-01	8.5E-06
Mmp19	1.5E-05	2.0E-09	2.8E-06	1.5E-42
Timp1	4.1E-18	1.1E-22	3.1E-15	1.1E-95

Figure S1. Top canonical pathways following infection with VACV-Wyeth and VACV-WR. IPA analysis of brain gene expression 2, 4 and 5 d.p.i with VACV-Wyeth (106 pfu) and VACV-WR (100pfu). Shared pathways are color coded respectively.

**No.**	**VACV-Wyeth (p-values)**				**No.**	**VACV-WR (p-values)**			
#	**Canonical Pathway**	**Day2**	**Day4**	**Day5**	#	**Canonical Pathway**	**Day2**	**Day4**	**Day5**
1	Th1 and Th2 Activation Pathway	0.5	23.0	34.4	1	Hepatic Fibrosis / Hepatic Stellate Cell Activation	0.5	19.6	23.0
2	Th1 Pathway	0.5	16.7	30.2	2	Granulocyte Adhesion and Diapedesis	0.0	13.9	19.2
3	T Helper Cell Differentiation	1.9	16.3	28.5	3	Role of Macrophages Fibroblasts and Endothelial Cells in Rheumatoid Arthritis	1.4	11.4	20.0
4	Type I Diabetes Mellitus Signaling	2.3	15.2	24.1	4	Atherosclerosis Signaling	0.0	12.6	19.5
5	Th2 Pathway	0.5	16.3	24.3	5	Acute Phase Response Signaling	1.3	14.0	16.4
6	TREM1 Signaling	0.0	14.4	24.7	6	Role of Pattern Recognition Receptors in Recognition of Bacteria and Viruses	0.5	9.4	19.0
7	Role of Pattern Recognition Receptors in Recognition of Bacteria and Viruses	2.4	16.2	20.5	7	Agranulocyte Adhesion and Diapedesis	0.0	14.2	14.4
8	Role of Macrophages Fibroblasts and Endothelial Cells in Rheumatoid Arthritis	2.7	13.1	22.3	8	Dendritic Cell Maturation	0.6	8.1	17.1
9	Altered T Cell and B Cell Signaling in Rheumatoid Arthritis	0.5	13.2	24.2	9	Type I Diabetes Mellitus Signaling	0.0	7.0	17.8
10	iCOS-iCOSL Signaling in T Helper Cells	1.1	14.5	21.6	10	Th1 Pathway	0.0	7.1	16.5

11	Dendritic Cell Maturation	2.7	13.1	20.3	11	Colorectal Cancer Metastasis Signaling	2.8	6.6	13.9
12	Acute Phase Response Signaling	2.5	14.9	16.4	12	Th1 and Th2 Activation Pathway	0.0	5.8	17.3
13	Hepatic Fibrosis / Hepatic Stellate Cell Activation	2.4	11.2	18.4	13	Glucocorticoid Receptor Signaling	7.5	6.2	9.2
14	EIF2 Signaling	18.8	12.8	0.0	14	Role of Osteoblasts Osteoclasts and Chondrocytes in Rheumatoid Arthritis	1.0	6.4	14.4
15	CD28 Signaling in T Helper Cells	1.4	12.4	17.5	15	Axonal Guidance Signaling	5.9	6.0	9.6
16	Role of NFAT in Regulation of the Immune Response	2.6	13.1	15.4	16	LXR/RXR Activation	0.0	8.7	12.8
17	Antigen Presentation Pathway	6.3	9.7	14.9	17	TREM1 Signaling	0.0	5.1	15.9
18	PKCθ Signaling in T Lymphocytes	1.8	11.7	15.8	18	IL-8 Signaling	4.6	3.5	12.7
19	Crosstalk between Dendritic Cells and Natural Killer Cells	0.5	11.6	17.1	19	IL-10 Signaling	1.0	7.1	12.0
20	Death Receptor Signaling	2.3	9.5	16.3	20	Molecular Mechanisms of Cancer	6.7	3.0	10.4
21	Communication between Innate and Adaptive Immune Cells	0.0	10.7	17.0	21	T Helper Cell Differentiation	0.0	4.8	15.1
22	IL-10 Signaling	2.1	12.5	12.1	22	NF-κB Signaling	2.5	4.1	12.7
23	Tec Kinase Signaling	1.4	9.4	15.1	23	Production of Nitric Oxide and Reactive Oxygen Species in Macrophages	1.8	6.3	11.2
24	Granulocyte Adhesion and Diapedesis	0.0	10.5	15.3	24	Osteoarthritis Pathway	1.7	6.5	10.7
25	Atherosclerosis Signaling	0.3	10.1	15.1	25	Altered T Cell and B Cell Signaling in Rheumatoid Arthritis	0.0	3.1	15.6
26	T Cell Receptor Signaling	1.5	11.0	12.9	26	Antigen Presentation Pathway	0.3	7.8	10.5
27	IL-6 Signaling	2.0	8.6	14.0	27	Th2 Pathway	0.3	3.7	14.2
28	Phagosome Formation	1.4	9.7	13.2	28	EIF2 Signaling	18.1	0.0	0.0
29	NF-κB Signaling	2.0	7.9	13.9	29	IL-6 Signaling	0.9	6.3	10.5
30	PI3K Signaling in B Lymphocytes	2.7	10.6	10.3	30	Leukocyte Extravasation Signaling	1.5	6.9	9.2
31	Interferon Signaling	5.3	7.5	10.9	31	Phagosome Formation	0.9	3.9	11.8
32	Graft-versus-Host Disease Signaling	1.1	9.4	12.4	32	Death Receptor Signaling	0.3	5.7	10.1
33	Production of Nitric Oxide and Reactive Oxygen Species in Macrophages	1.8	8.5	11.9	33	Communication between Innate and Adaptive Immune Cells	0.0	2.4	13.6
34	Toll-like Receptor Signaling	1.4	8.2	12.6	34	iCOS-iCOSL Signaling in T Helper Cells	0.7	1.5	13.5

Figure S2. Antigen Presentation Pathway. Genes associated with antigen presentation following infection with (A) VACV-Wyeth (-log(*p*-values): 6.3) and (B) VACV-WR 2 d.p.i. (-log(*p*-values): 0.3). Differentially expressed genes are highlighted in color. Color intensity indicates the degree of up regulation (red) or down-regulation (green) relative to the day 2 mock infected brains. Solid lines represent direct interactions. (For interpretation of the references to color in this figure legend, the reader is referred to the web version of this article).

A

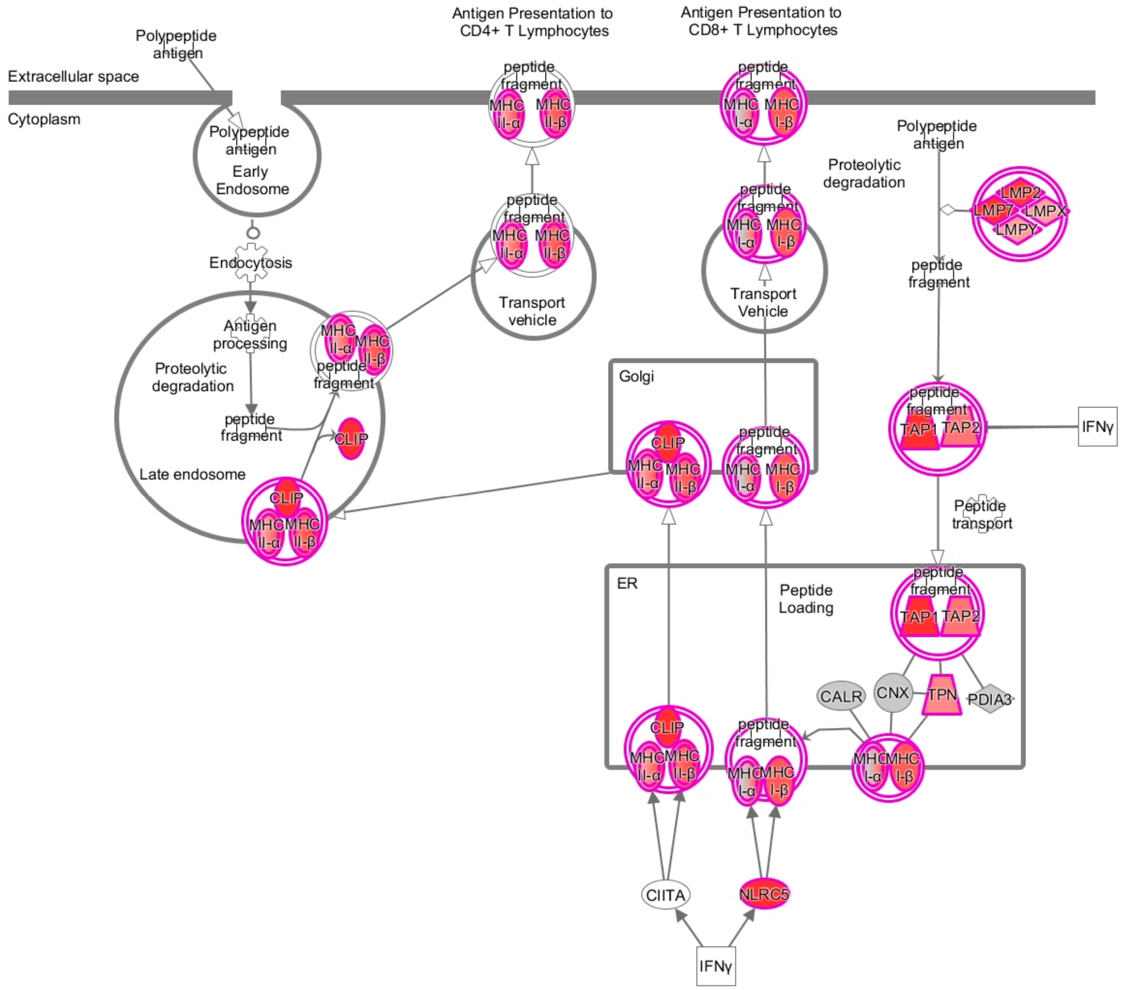

B

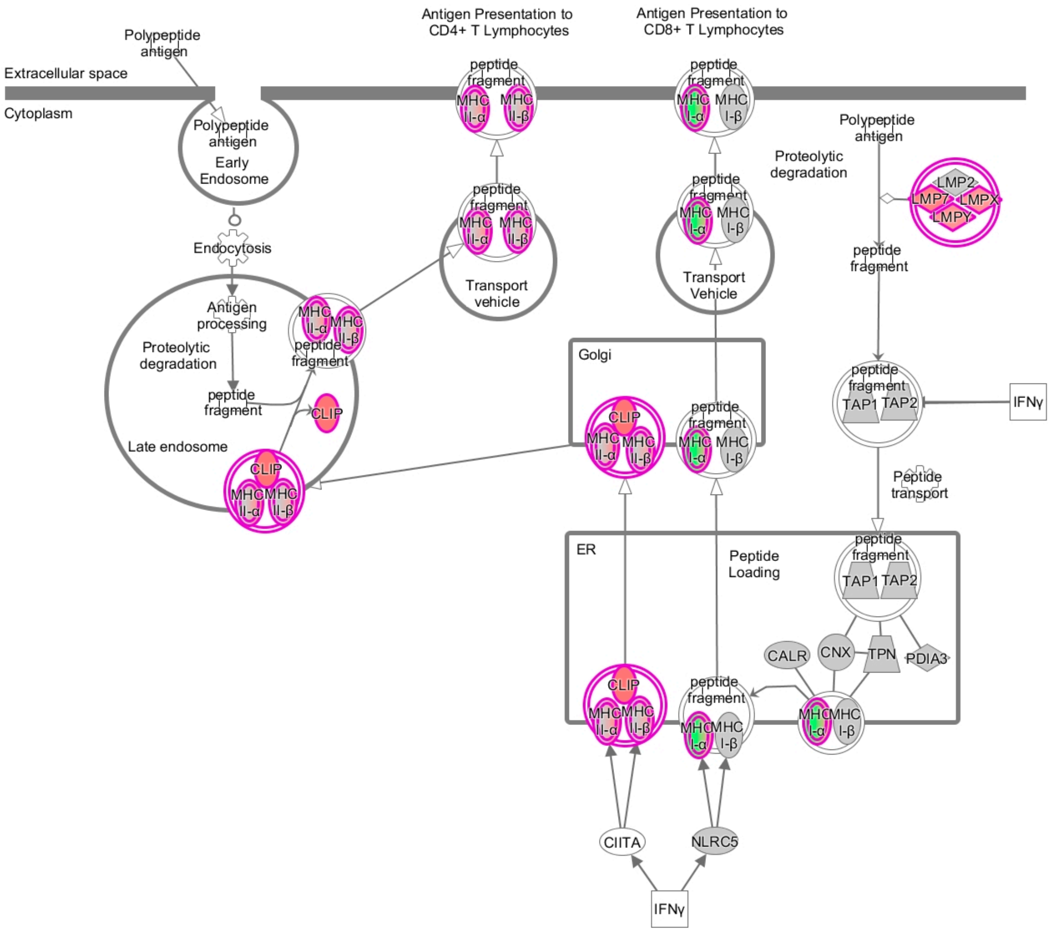
Polypeptide antigen
Extracellular space
Cytoplasm
Antigen Presentation to CD4+ T Lymphocytes
Antigen Presentation to CD8+ T Lymphocytes
peptide fragment
MHC II-α
MHC II-β
MHC I-α
MHC I-β
Polypeptide antigen
Early Endosome
Endocytosis
Proteolytic degradation
LMP2
LMP7
LMPX
LMPY
Transport vehicle
Transport Vehicle
Antigen processing
Proteolytic degradation
CLIP
Late endosome
Golgi
TAP1
TAP2
IFNγ
Peptide transport
ER
Peptide Loading
CALR
CNX
TPN
PDIA3
CIITA
NLRC5
IFNγ

Figure S3. Mitochondrial Dysfunction Pathway. Genes associated with mitochondrial dysfunction following infection with (A) VACV-Wyeth (-log(*p*-values): 6.6) and (B) VACV-WR 2 d.p.i. (-log(*p*-values): 13.7). Differentially expressed genes are highlighted in color. Color intensity indicates the degree of up regulation (red) or down-regulation (green) relative to the day 2 mock infected brains. Solid lines represent direct interactions and dashed lines represent indirect interactions. (For interpretation of the references to color in this figure legend, the reader is referred to the web version of this article).

A

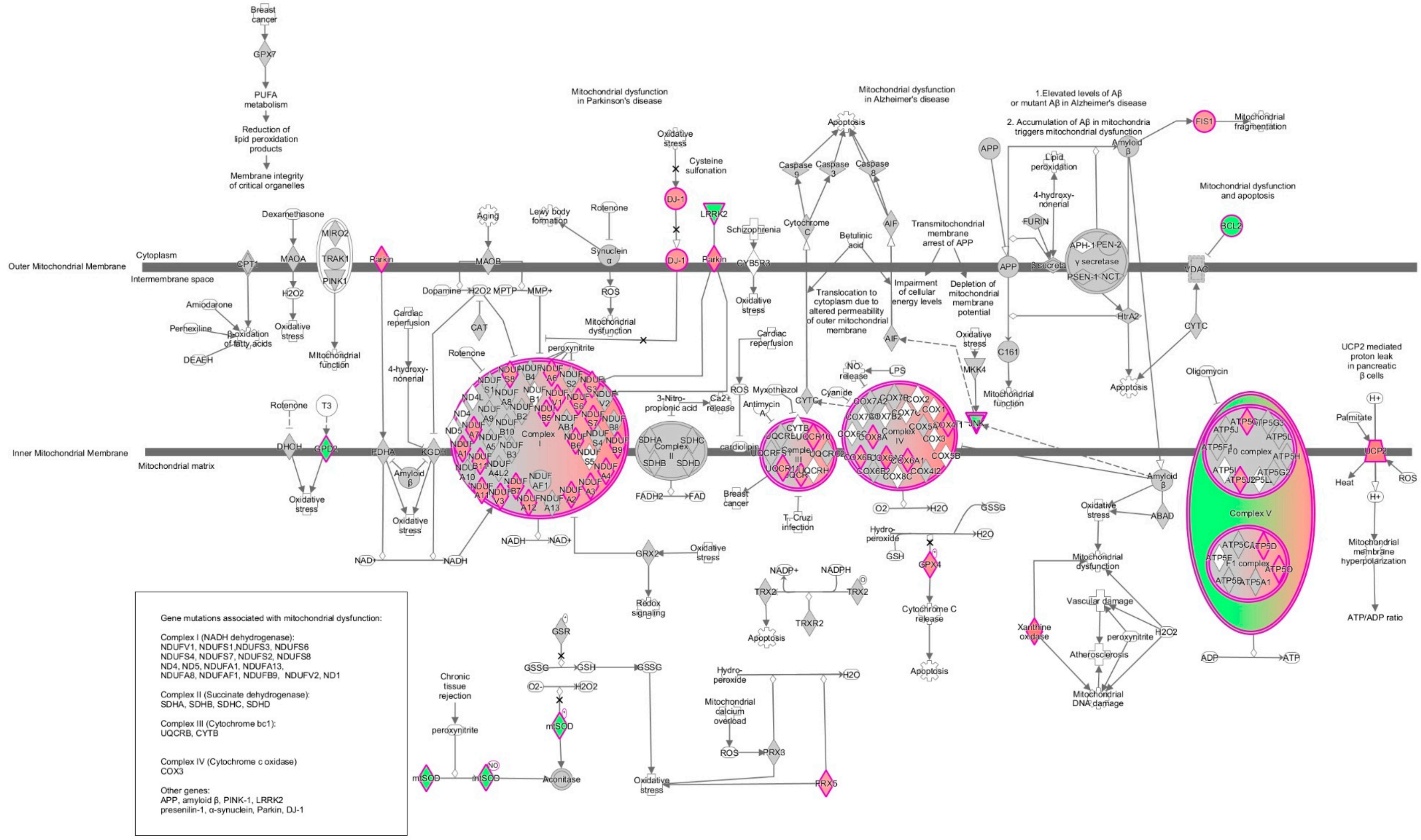

B

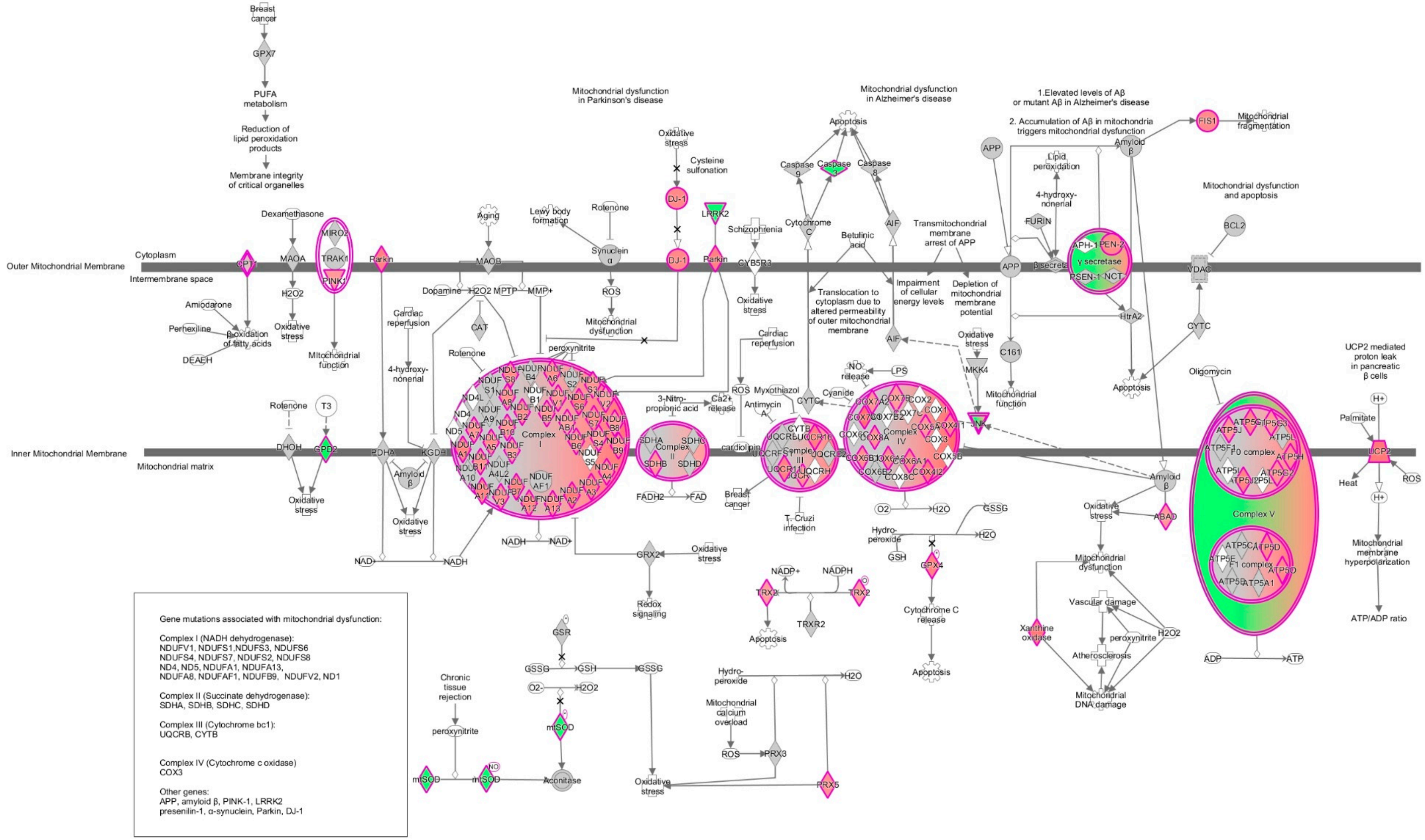
Outer Mitochondrial Membrane
Cytoplasm
Intermembrane space
Inner Mitochondrial Membrane
Mitochondrial matrix
Mitochondrial dysfunction
in Parkinson's disease
Mitochondrial dysfunction
in Alzheimer's disease
1.Elevated levels of Aβ
or mutant Aβ in Alzheimer's disease
2. Accumulation of Aβ in mitochondria
triggers mitochondrial dysfunction
Mitochondrial dysfunction
and apoptosis
UCP2 mediated
proton leak
in pancreatic
β cells
Breast
cancer
GPX7
PUFA
metabolism
Reduction of
lipid peroxidation
products
Membrane integrity
of critical organelles
Dexamethasone
MAOA
MAOB
CPT1
MIRO2
TRAK1
PINK1
Parkin
DJ-1
LRRK2
Oxidative
stress
Cysteine
sulfonation
Schizophrenia
CYB5R3
Synuclein
α
Lewy body
formation
Rotenone
Aging
Dopamine
H2O2
MTPP
MMP+
CAT
ROS
Mitochondrial
dysfunction
peroxynitrite
Cardiac
reperfusion
4-hydroxy-
nonenal
Amiodarone
Perhexiline
DEAEH
β-oxidation
of fatty acids
Mitochondrial
function
Apoptosis
Caspase
9
Caspase
3
Caspase
8
Cytochrome
C
AIF
Betulinic
acid
Transmitochondrial
membrane
arrest of APP
Translocation to
cytoplasm due to
altered permeability
of outer mitochondrial
membrane
Impairment
of cellular
energy levels
Depletion of
mitochondrial
membrane
potential
APP
Amyloid
β
Lipid
peroxidation
4-hydroxy-
nonenal
FURIN
APH-1
PEN-2
γ secretase
PSEN-1
NCT
HtrA2
FIS1
Mitochondrial
fragmentation
BCL2
VDAC1
CYTC
C161
MKK4
JNK
LPS
NO
release
Cyanide
Myxothiazol
Antimycin
A
3-Nitro-
propionic acid
Ca2+
release
cardiolipin
Complex
I
Complex
II
Complex
III
Complex
IV
SDHA
SDHB
SDHC
SDHD
FADH2
FAD
Breast
cancer
T. Cruzi
infection
O2
H2O
GSSG
Hydro-
peroxide
GSH
GPX4
Cytochrome C
release
Oligomycin
F0 complex
F1 complex
Complex V
ADP
ATP
H+
Palmitate
UCP2
Heat
ROS
Mitochondrial
membrane
hyperpolarization
ATP/ADP ratio
Amyloid
β
ABAD
Oxidative
stress
Mitochondrial
dysfunction
Vascular damage
peroxynitrite
H2O2
Atherosclerosis
Xanthine
oxidase
Mitochondrial
DNA damage
Rotenone
T3
DHOH
GPD2
PDHA
KGDH
Amyloid
β
Oxidative
stress
NAD+
NADH
GRX2
Redox
signaling
NADP+
NADPH
TRX2
TRXR2
Apoptosis
GSR
GSSG
GSH
O2-
mtSOD
Chronic
tissue
rejection
peroxynitrite
Aconitase
Hydro-
peroxide
H2O
Mitochondrial
calcium
overload
ROS
PRX3
PRX5
Oxidative
stress
Gene mutations associated with mitochondrial dysfunction:
Complex I (NADH dehydrogenase):
NDUFV1, NDUFS1,NDUFS3, NDUFS6
NDUFS4, NDUFS7, NDUFS2, NDUFS8
ND4, ND5, NDUFA1, NDUFA13,
NDUFA8, NDUFAF1, NDUFB9, NDUFV2, ND1
Complex II (Succinate dehydrogenase):
SDHA, SDHB, SDHC, SDHD
Complex III (Cytochrome bc1):
UQCRB, CYTB
Complex IV (Cytochrome c oxidase)
COX3
Other genes:
APP, amyloid β, PINK-1, LRRK2
presenilin-1, α-synuclein, Parkin, DJ-1

Figure S4. Oxidative Phosphorylation Pathway. Genes associated with oxidative phosphorylation following infection with (A) VACV-Wyeth (-log(*p*-values): 7.0)and (B) VACV-WR 2 d.p.i. (-log(*p*-values): 14.2). Differentially expressed genes are highlighted in color. Color intensity indicates the degree of up regulation (red) or down-regulation (green) relative to the day 2 mock infected brains. Solid lines represent direct interactions and dashed lines represent indirect interactions. (For interpretation of the references to color in this figure legend, the reader is referred to the web version of this article).

A

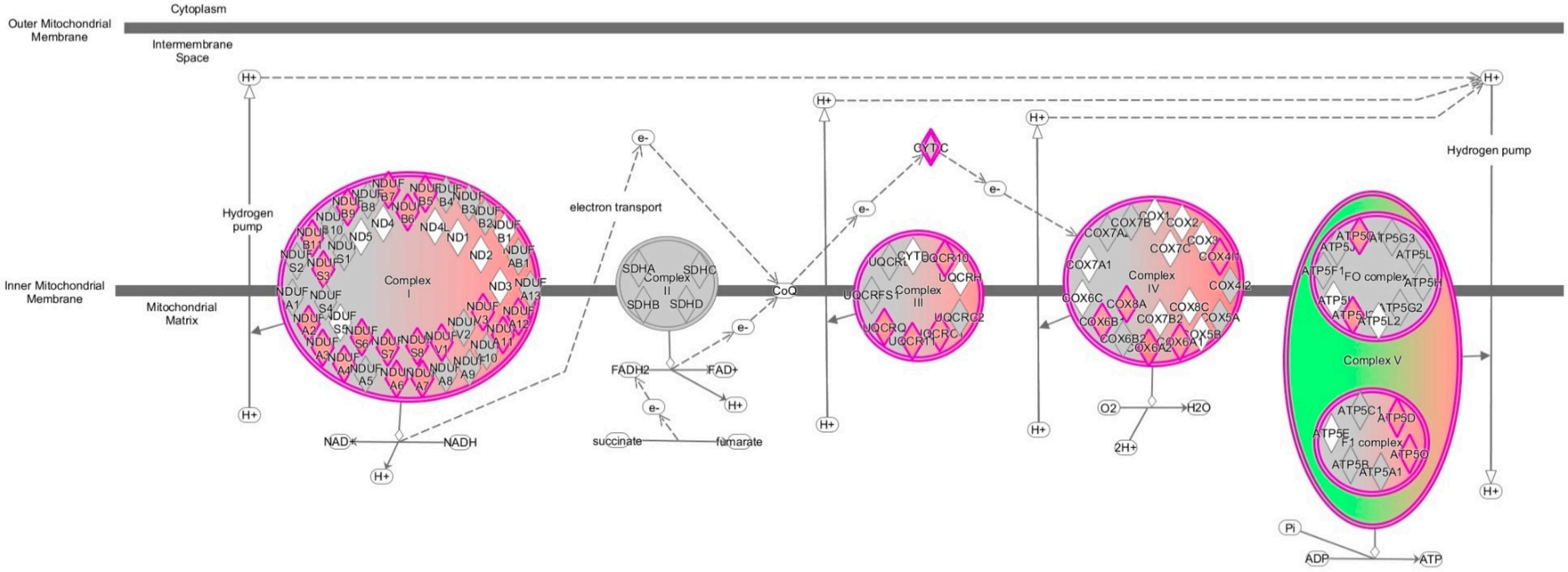

B

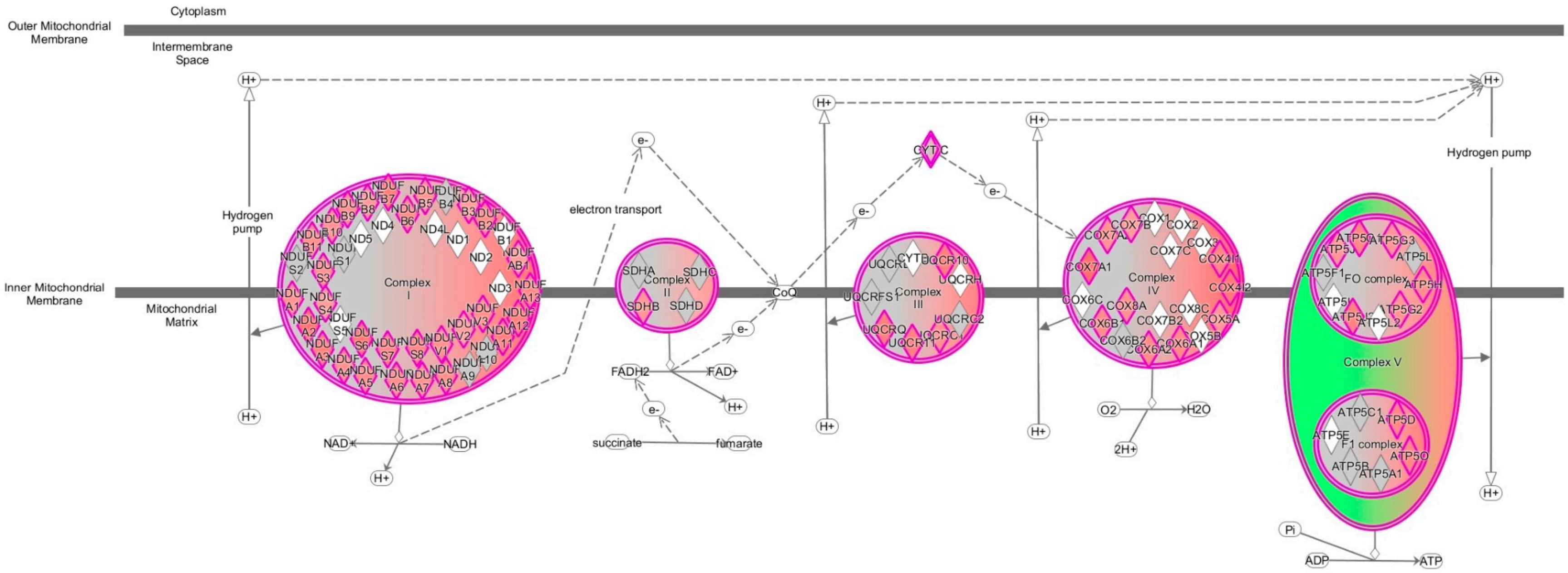
Cytoplasm
Outer Mitochondrial Membrane
Intermembrane Space
Inner Mitochondrial Membrane
Mitochondrial Matrix
Hydrogen pump
electron transport
Complex I
Complex II
Complex III
Complex IV
Complex V
FO complex
F1 complex
Hydrogen pump
NAD+
NADH
FADH2
FAD+
succinate
fumarate
CoQ
CYT C
O2
H2O
2H+
Pi
ADP
ATP
